# Polycyclic Aromatic Hydrocarbons (PAHs) in the Dissolved Phase, Particulate Matter, and Sediment of the Sele River, Southern Italy: A Focus on Distribution, Risk Assessment, and Sources

**DOI:** 10.3390/toxics10070401

**Published:** 2022-07-19

**Authors:** Paolo Montuori, Elvira De Rosa, Fabiana Di Duca, Bruna De Simone, Stefano Scippa, Immacolata Russo, Pasquale Sarnacchiaro, Maria Triassi

**Affiliations:** 1 Department of Public Health, “Federico II” University, Via Sergio Pansini no 5, 80131 Naples, Italy; elvira_derosa@libero.it (E.D.R.); fabianadiduca91@gmail.com (F.D.D.); desimonebruna7@gmail.com (B.D.S.); stefanoscippa923@gmail.com (S.S.); imrusso@unina.it (I.R.); triassi@unina.it (M.T.); 2 Department of Law and Economics, “Federico II” University, Via Cinthia 26, 80126 Naples, Italy; sarnacch@unina.it

**Keywords:** polycyclic aromatic hydrocarbons, Sele River, fugacity, source, TEQ

## Abstract

The Sele River, located in the Campania Region (southern Italy), is one of the most important rivers and the second in the region by average water volume, behind the Volturno River. To understand the distribution and sources of polycyclic aromatic hydrocarbons (PAHs) in the Sele River, water sediment samples were collected from areas around the Sele plain at 10 sites in four seasons. In addition, the ecosystem health risk and the seasonal and spatial distribution of PAHs in samples of water and sediment were assessed. Contaminant discharges of PAHs into the sea were calculated at about 1807.9 kg/year. The concentration ranges of 16 PAHs in surface water (DP), suspended particulate matter (SPM), and sediment were 10.1–567.23 ng/L, 121.23–654.36 ng/L, and 331.75–871.96 ng/g, respectively. Isomeric ratio and principal component analyses indicated that the PAH concentrations in the water and sediment near the Sele River were influenced by industrial wastewater and vehicle emissions. The fugacity fraction approach was applied to determine the trends for the water-sediment exchange of 16 priority PAHs; the results indicated that fluxes, for the most part, were from the water into the sediment. The toxic equivalent concentration (TEQ) of carcinogenic PAHs ranged from 137.3 to 292.6 ngTEQ g^−1^, suggesting that the Sele River basin presents a definite carcinogenic risk.

## 1. Introduction

Polycyclic aromatic hydrocarbons (PAHs) are a class of ubiquitous and persistent pollutants that are highly dangerous for humans, as they are carcinogenic and mutagenic. The extent of the potential risk and the distribution of PAHs in the environment is a public health issue [1,2]. With population development and economic growth, the input of PAHs intensified considerably in the 20th century; therefore, 16 PAHs have been identified as priority contaminants by the U.S. Environmental Protection Agency [3]. Seven of them, namely benz[a]anthracene, chrysene, benzo[b]fluoranthene, benzo[k]fluoranthene, benzo[a]pyrene, indo[1,2,3-cd]pyrene, and dibenzo[a,h]anthracene, are potentially carcinogenic to humans according to the International Agency for Research on Cancer [4,5]. In addition, four PAHs (benzo[a]pyrene, benz[a]anthracene, benzo[b]fluoranthene and chrysene) were recently defined as the main indicators of the presence of genotoxic and mutagenic PAHs in the environment and, in particular, in food [6]. The majority of the PAH load in the environment is from the combustion of organic matter (pyrolytic origin), which is usually released from human activities, such as coal combustion, petrol and diesel oil combustion, industrial processes, and home heating. Other types of non-anthropogenic sources, such as petrogenic and diagenetic origins, are relatively less abundant [7,8]. PAHs introduced into the aquatic environment move from the water into the sediment due to their chemical and physical properties; in particular, the high-molecular-weight PAHs, consisting of several aromatic rings, have a greater tendency to bind to the sediment. In contrast, low-molecular-weight (LMW) PAHs, consisting of few aromatic rings, degrade faster, and their concentrations in surface water and sediments are relatively low [9]. The partitioning of PAHs in water and sediment is one of the major processes controlling the toxicity of PAHs in aquatic environments [10]. Over the years, numerous studies have been conducted on important rivers in central and southern Italy to evaluate and estimate the PAH levels in the water, suspended particulate matter, and sediment; toxicity was also assessed to verify the harmful effects on the environment and the possible biological risks for living organisms in the watercourses.

Beginning with central Italy, the Tiber River has concentration ranges of 10.3 to 951.6 ng/L (DP + SPM) and 36.2 to 545.6 ng/g for the sediment, with a relatively low toxicity [11]. In southern Italy, we find the Volturno River, with concentration ranges from 256.0 to 1686.3 ng/L (DP + SPM) and 434.8 to 872.1 ng/g for the sediment, with a toxicity value that highlights an area possibly at risk [12]. In the Sarno River, on the other hand, ranges of 23.1 to 2670.4 ng/L (DP + SPM) and 5.3 to 678.6 ng/g for sediment are found, with toxicity values that do not indicate an area experiencing immediate biological effects [13]. Qu et al. [14] studied the Gulfs of Salerno and Naples, reporting concentrations for the sediment from 9.58 to 15.81 µg/kg for the Bagnoli area, 317 µg/kg for the Salerno area, and 768.0 µg/kg for the Gulf of Naples area, with significant toxicity and biological risk values. Campania is one of the most populated regions of Italy, with over half of its population concentrated in metropolitan areas such as Naples and Salerno. Currently, industrial activity, agricultural practices, and illegal waste disposal represent difficult problems in the effort to mitigate the high levels of contamination in the Campania plain [14,15]. The plain is dominated by the presence of numerous industrial activities, including dairies, canning, and chemical industries. In addition, there are many contaminated sites, both landfills and illegal disposal areas. There are also well-developed agricultural activities in the region, such as livestock farming (buffalo farms); the large-scale production of vegetables and fruits feeds the local food industry. In areas where mainly agricultural and livestock products are processed, the emission of waste with high amounts of organic and inorganic substances can impact ecological and environmental integrity [13,16]. The Gulf of Salerno is one of the main environments in which pollutants accumulate from the Campania Plain. The Sele River is an important river in the Campania region; it has a length of 64 km and is the second in the region and the south of Italy by average water volume, behind the Volturno River.

The current paper reports the concentrations of PAHs in the water and sediment of the Sele River in the Gulf of Salerno (central Mediterranean Sea), southern Italy. The specific objectives of the present study are to: (I) investigate the contamination levels and spatial distribution of PAHs, (II) identify their potential sources, and (III) estimate the environmental risk in this area.

## 2. Materials and Methods

### 2.1. Study Area

**Figure 1:**
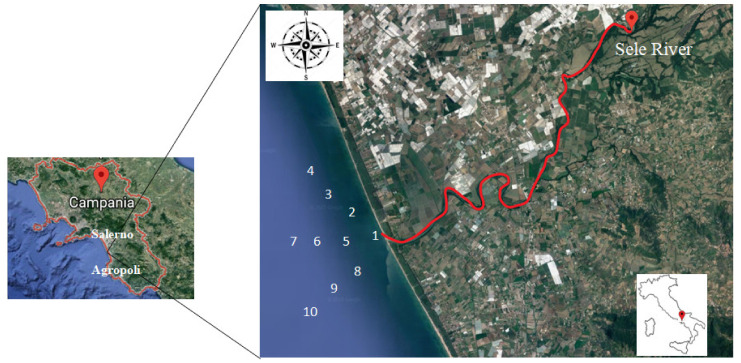
Map of the study area and sampling sites along the Sele River and estuary, southern Italy.

The Sele River basin (3236 km^2^) is located on the western (i.e., Tyrrhenian) side of southern Italy and includes a large alluvial plain. The plain has a triangular surface area of about 400 km^2^. It is delimited offshore by a narrow sandy coastal strip between the towns of Salerno (NW) and Agropoli (SE); landward, it is delimited to the north and northwest by the Lattari and Picentini Mountains and to the southeast by the Alburni Mountains and the Cilento Promontory (Figure 1) [17]. The climate in the Sele basin is of Mediterranean type, with important spatial variations in both erosive rainfall and temperature according to the elevation and the distance from the coast. The Mediterranean climate is characterized by mild temperatures. It is a particularly dry climate in summer and mild in winter. Rainfall is concentrated from autumn to spring, and in the driest month of the year is less than 30 mm, which is about a third of the wettest month. The lack of rainfall in the summer, with at least two consecutive months of drought, is a peculiarity of the Mediterranean climate. In other climate classifications, precipitation is concentrated in the hot season. In the Mediterranean climate, the sea contributes to determining the climate, which is warm temperate, with modest daily and annual temperature ranges (less than 21 °C); in fact, the sea retains the summer heat, accumulating and then releasing it during the winter. The combination of dry summers and rainy winters is a typical characteristic of the Mediterranean climate.

An increase in population density, high industrial pollution, the presence of road and railway networks, and an increasing influx of tourists to the city of Salerno have caused an increase in environmental pollution in this area [18].

### 2.2. Sampling

A total of 40 surface water samples and 10 sediment samples were collected in the summer, autumn, winter, and spring of 2020–2021 from 10 sampling locations along the Sele River (Table 1). For each season and at each sampling point, three sample aliquots were taken. This process was repeated in duplicate. The aliquots were transported to the laboratory and analyzed in triplicate to calculate the standard deviation and evaluate the repeatability of the method.

The first sampling point was the river mouth, with the purpose of assessing downstream pollution; in addition, nine other points were sampled at 500, 1000, and 1500 mt away from the river mouth to evaluate the impact of Sele River pollution on the Mediterranean Sea environment (Figure 1). The samples were collected in 2.5 L amber bottles, using 6 M of hydrochloric acid, from the surface layer at a depth of 0–50 cm from the sampling locations, while sediment samples were collected at 0–5 cm with a Van Veen Grab sampler and preserved in aluminum boxes. All samples were temporarily stored in refrigerated containers containing crushed ice until they were transported back to the laboratory and preserved at −20 °C until analysis.

### 2.3. Extraction and Analysis

The samples collected were transported to the laboratory within 24 h, and they were filtered through 47 mm × 0.7 µm glass fiber filters (Whatman, Maidstone, UK) that had been heated at 400 °C overnight to separate the water from the suspended particulate matter (SPM). The dissolved phase PAHs were extracted from water samples using a solid-phase extraction (SPE) cartridge by Oasis HLB (6 mL, 500 mg; Waters, Milford, MA, USA), according to the method proposed by Liu et al. [19]. C18 SPE cartridges (to elute and concentrate) were pre-washed with dichloromethane (DCM) before conditioning with methanol and ultrapure water; then, 10 μL of surrogate standard (benzo[a]pyrene-d_12_ and indeno[1,2,3-cd]pyrene-d_12_) was added to 1 L of the water sample before mixing. Following that, the water sample was passed through a column for concentration at a flow rate of 3 mL/min. Next, a vacuum pump was used to dry the column. The eluate was concentrated to 0.5 mL using a nitrogen flow before 10 μL of internal standard (Chrysene-d_12_) was added, followed by GC/MS analysis.

SPM content was determined by gravimetry. First, the filter was dried in an air-heated oven (55 °C until constant weight) and equilibrated at room temperature in a desiccator. Filters were then spiked with three surrogate standards (10 ng of chrysene-d12, benzo[a]pyrene-d12, and indeno[1,2,3-cd]pyrene-d12) and extracted three times by sonication with 10 mL of dichloromethane-methanol (1:1) for 15 min. After extraction, the extracts were concentrated using a rotary evaporator. The volume of the extracts was adjusted to 0.5 mL and solvent-exchanged into hexane. Cleanup and fraction procedures were performed with open column chromatography (3 g of neutral alumina deactivated with 3% (*w*/*w*) Milli-Q water). Three fractions were collected: fraction I with 5.5 mL of hexane, fraction II with 6 mL of hexane:ethylacetate (9:1), and fraction III with 12 mL of ethylacetate. PAHs were eluted in fraction II, while fractions I and III contained other organic pollutants that were also detected in the samples. The sediment samples were air-dried in the dark for 5 days, crushed, sieved (250 µm particles were used as the sample), and divided into 5 g portions. The PAH concentrations in the sediment samples were calculated according to dry weight (ng/g dw) [20,21]. PAHs were extracted from the filters and sediment samples using a Soxhlet extractor (Table S1). As in [22], the samples were draped onto a filter paper, placed into the cellulose extraction thimble, and covered with cotton wool. The thimble was located inside the main Soxhlet chamber and fitted to a 250 mL round-bottomed flask containing methylene chloride (150 mL). A condenser was then attached. The samples were extracted for 24 h under reflux. The extracts were purified through a column composed of 1 g of sodium sulfate and 2.5 g (10% deactivated) of silica gel and eluted with 70 mL of a hexane:methylene chloride (7:3) solution. The extracts were evaporated to dryness, reduced to a final volume (500 μL) using flushing nitrogen gas, and chrysene-d_12_ was added as an internal standard. To evaluate the organic carbon normalized partition coefficients (Koc’), which estimate PAH attraction to sediment and define the sediment-water partitioning level, the total organic carbon (TOC) content of the sediments was analyzed using a TOC analyzer (TOC-VCPH, Shimadzu Corp., Kyoto, Japan).

### 2.4. Instrumental Analysis

All samples were analyzed on a gas chromatograph with a mass spectrometer detector (TRACE^TM^ 1310 Gas Chromatograph coupled to an ISQ^TM^ 7000 Single Quadrupole Mass Spectrometer, Thermo Scientific, Waltham, MA, USA) to determine PAHs with selected ion monitoring (SIM) (Table S2). A TG-5MS capillary column with 30 mm length × 0.25 mm inner diameter × 0.25 μm film thickness was used. The column temperature was programmed to rise from 60 °C to 200 °C for 2 min at 25 °C min^−1^, then to 270 °C at 10 °C min^−1^ (maintained for 6 min), and finally, to 310 °C at 25 °C min^−1^ (maintained for 10 min). The mass spectrometer was operated in the electron ionization (EI) mode set at 70 eV, and the injector and detector temperatures were 280 °C and 300 °C, respectively (Table S3). Acquisition was carried out in the single ion monitoring mode (SIM) using two characteristic ions for each target analyte. Target analytes were identified and verified by comparing the retention times of the samples with standards and using the characteristic ions and their ratios for each target analyte. Furthermore, for the more highly concentrated samples, the identification of target analytes was confirmed in full-scan mode (*m*/*z* range from 60 to 350), and the analytes were quantified using the characteristic ions and their ratios for each target analyte. The concentrations of 16 PAHs were determined: naphthalene (Nap), acenaphthene (Ace), acenaphthylene (Acy), fluorine (Flu), phenanthrene (Phe), anthracene (Ant), fluoranthene (Fla), pyrene (Pyr), benz[a]anthracene (BaA), chrysene (Chr), benzo[b]fluoranthene (BbF), benzo[k]fluoranthene (BkF), benzo[a]pyrene (BaP), dibenz[a,h]anthracene (DahA), indeno[1,2,3-cd]pyrene (IcdP), and benzo[g,h,i]perylene (BghiP). PAH quantification was performed using a five-point calibration curve (5–25–100–500–1000 ng/L) for the 16 PAHs (Dr. Ehrenstorfer GmbH, Augsburg, Germany) (r^2^ > 0.97), and chrysene-d_12_ was used as an internal standard. The quantification of individual compounds was determined by the comparison of peak areas with those of the recovery standards. The samples were analyzed in triplicate. For the water-dissolved phase samples (final concentration in water of 10 ng L^−1^), after passage through a column, the eluate was concentrated to 0.5 mL using a nitrogen flow before 10 µL of internal standard (chrysene_d12_) was added.

The PAH concentrations in the sediment samples were calculated according to dry weight (ng/g dw). PAHs were extracted from the filters and sediment samples using a Soxhlet extractor. The samples were extracted for 24 h under reflux. The extracts were evaporated to dryness, reduced to a final volume (500 µL) using flushing nitrogen gas, and chrysene_d12_ was added as an internal standard.

The detection limit (LOD) was calculated as three times the noise in a blank sample chromatogram. In the water and SPM, LODs ranged from 1.3 to 1.6 ng L^−1^; in sediment samples, they ranged from 1.5 to 1.9 ng g^−1^. The quantification limits (LOQ) were in the range of 4.8–5.4 ng L^−1^ in the water and SPM samples and 5.1–6.3 ng g^−1^ in the sediment samples (Tables S4 and S5). A total of ten blanks were analyzed in the same manner as the samples; the PAHs in the blanks showed a concentration below the LOD. The recovery of PAHs in the standard checks and samples was between 70% and 130%, which met quality control requirements. For the effective and reproducible detection and quantification of low concentrations of PAHs in water, several parameters were determined, such as linear range (5–25–100–500–1000 ng/L), precision, limit of detection, and limit of quantification. The precision of the method was determined through repeatability studies and was expressed as relative standard deviation (RSD). The average of the results was used to estimate the precision of the method. The RSD was determined by analyzing one sample on the same day, with the same instrument, and by the same analyst under identical conditions.

### 2.5. Water-Sediment Partitioning

Water-sediment partitioning is an important environmental process that can be used to evaluate the equilibrium partition behavior of PAHs in aquatic environments [10,19]. The K_ow_ (octanol-water partition coefficient) is the coefficient expressing the lipophilicity or carbon affinity of a chemical, and it is related to the distribution coefficient so as to describe the fate of environmental pollutants such as PAHs [10]. Organic carbon normalized partition coefficients (Koc) estimate PAH attraction to sediment and define the sediment-water partitioning level [23,24,25]. In order to assess the behavior of PAHs in the Sele River area, in situ organic carbon coefficients (*K_oc_′*) were calculated by Equation (1) [26,27]:

*K_oc_′* = *C_S_*/(*C_aq_* × *f_oc_*) (1)

where *C_s_* and *C_aq_* are the PAH concentrations in the solid and liquid phases, respectively, and *f_oc_* is the percentage of organic carbon in the sediment.

The difference between log *K_oc_′* and the corresponding log *K_oc_* indicates the equilibrium state of PAHs in an aquatic system [28].

If the average log *K_oc_′* is lower than the corresponding *K_oc_* and *K_ow_*, PAHs are more absorbed into the sediment phase than exchanged into the water phase [27].

The movement of a chemical from one area to another is monitored by fugacity. For this reason, the exchange processes of PAHs between water and sediment were estimated by the fugacity fraction [2,29]. In Equation (2), *ff* is defined:

*ff* = *K_oc_′*/(*K_oc_′* + *K_oc_*) (2)

A value of *ff* < 0.3 indicates that PAHs are adsorbed into the sediment from water and that sediments act as a sink for PAHs. Values in the range 0.3 < *ff* < 0.7 describe sediment-water equilibrium, and when *ff* ˃ 0.7, a flux from sediment to water is predicted, and sediments act as a secondary emission source of PAHs [19,28].

### 2.6. Risk Assessment and Determination of Toxicity

#### 2.6.1. Biological Adverse Effects

In sediment, PAHs can be very dangerous to life in the aquatic ecosystem and a source of pollutants that accumulate in the food chain [29].

In this study, the sediment quality guidelines (SQGs) were used to estimate the potentially toxic effects of contaminants in the sediment samples on animals and marine organisms [30].

The SQGs estimate the toxicity that these contaminants cause to the aquatic environment based on the following ranges: effects range low (ERL)/effects range median (ERM) and threshold effects level (TEL)/probable effects level (PEL) [31,32].

ERL and TEL classifications correspond to chemical amounts below which the probability of toxicity and other effects are low. In contrast, the ERM and PEL classifications represent a mid-range above which negative effects are likely to occur. ERL-ERM and TEL-PEL classifications represent a possible effects range within which adverse effects sometimes occur [13,33].

#### 2.6.2. Toxicity Determination

Marine sediments are considered a contaminant pool of PAHs, and the potential toxicity of PAHs, in particular carcinogenic PAHs (C-PAHs), in the aquatic environment may threaten human health [34]. This study evaluated the potential impact of C-PAHs based on BaP toxic equivalency factors (TEFs). The toxic equivalent quantity (TEQ) of ΣPAHs was determined through the following equation:

TEQ_PAHs_ = ∑_i_ TEF_i_ × C_PAHi_ (3)

where TEF_i_ (toxic equivalency factor) is the toxic factor of each carcinogenic PAH relative to BaP and C_PAHs_ and represents the concentration of an individual carcinogenic PAH.

The TEF_s_ values determined by the U.S. EPA [22] for each carcinogenic PAH are as follows: 0.1 for BaA, 0.001 for Chr, 0.1 for BbF, 0.01 for BkF, 1 for BaP, 0.1 for IcdP, and 1 for DahA [35].

### 2.7. Identifying the Source of PAHs

PAHs mainly derive from industrial processes and incomplete combustion by various industrial activities, such as waste incineration, iron and aluminum production, cement manufacturing, dye manufacturing, and asphalt industries, as well as from vehicle emissions and other anthropogenic activities [36,37].

Identifying the possible sources of PAH pollution is an important objective for the institutions seeking to collect information on how to control the pollution caused by these pollutants.

The sources of PAHs, whether from fuel combustion (pyrolytic) or from crude oil (petrogenic) contamination, may be determined by the ratios of specific PAH compounds based on peculiarities in PAH composition and distribution as a function of the emission source. The diagnostic ratios of selected PAHs were utilized to distinguish PAHs from pyrogenic and petrogenic sources. For example, HMW/LMW PAHs, Flu/(Flu + Pyr), IcdP/(IcdP + BghiP), BaA/(BaA + Chr), Ant/(Ant + Phe), and BbF/BkF were applied for PAH source identification [38,39].

LMW contaminants are more common in samples containing petrogenic PAHs, and HMW contaminants are common in samples containing pyrogenic PAHs; this is because most of the HMW molecules are formed at higher temperatures [40,41].

In this study, the principal component analysis (PCA) technique was used to quantitatively explore PAH origins. PCA was used as a multivariate analytical tool to reduce a set of original variables (measured PAH content in the sediment samples) and to extract a small number of latent factors (principal components, PCs) for analyzing relationships among the observed variables. As a result of an effective ordination process, the first PC accounts for the greatest proportion of the original variance, while the second and subsequent PCs progressively explain smaller amounts of data variation [42,43].

## 3. Results and Discussion

### 3.1. PAH Distribution in Water, SPM, and Sediment

Analysis of samples collected from the Sele River showed the presence of various PAHs in surface water, SPM, and sediment; the mean concentrations of ΣPAHs were 10.1–567.2 ng/L, 121.9–654.3 ng/L, and 331.7–871.1 ng/g, respectively (Table 1). The high anthropogenic pressure of the city of Salerno is evident in the presence of large food facilities and a vast industrial zone; in particular, the environment surrounding the Sele River is characterized by industrial districts, urban areas, intensive cultivations, and agricultural crops [44,45].

The concentrations of total PAHs in the water-dissolved phase (DP) detected at 10 locations along the Sele River and its estuary ranged from 3.4 to 98.5 ng L^−1^ for two-ring PAHs (Nap), from 22.7 to 164.2 ng L^−1^ for three-ring PAHs (Acy, Ace, Flu, Phe, and Ant), from 1.2 to 24.2 ng L^−1^ for four-ring PAHs (Fla, Pyr, BaA, and Chr), from 9.4 to 37.2 ng L^−1^ for five-ring PAHs (BbF, BkF, BaP, and DahA), and from 17.6 to 44.5 ng L^−1^ for six-ring PAHs (BghiP and IcdP) (Table S6).The compositional pattern of PAHs in the dissolved phase indicates that two- and three-ring PAHs were abundant at all sampling sites, representing, on average, over 60% of all PAHs. The predominance of low-molecular-weight PAHs (two-three-ring) in the water may be explained by their high water solubility and relatively high vapor pressures [46,47] (Figure S1).

The PAHs detected in SPM ranged from 3.2 to 62.3 ng L^−1^ for two-ring PAHs (Nap), from 21.2 to 58.3 ng L^−1^ for three-ring PAHs (Acy, Ace, Flu, Phe, and Ant), from 38.7 to 190.9 ng L^−1^ for four-ring PAHs (Fla, Pyr, BaA, and Chr), from 26.5 to 105.0 ng L^−1^ for five-ring PAHs (BbF, BkF, BaP, and DahA), and from 18.7 to 68.2 ng L^−1^ for six-ring PAHs (BghiP and IcdP) (Table S7). The compositional profiles of PAHs in SPM show that four-, five-, and six-ring PAHs were abundant at most sampling sites, accounting for 67% of ΣPAHs in SPM. Therefore, the higher PAH concentrations found in SPM may derive from PAH particles suspended in the air because the Sele River drainage basin passes through large agricultural areas and large industrial areas in southern Italy; these areas contain agri-food industries, chemical plants, and manufacturing industries. The emission of atmospheric particles from intensive agricultural activities and factories also causes serious air pollution, and the particulate-associated PAHs may be transported and deposited into the river [48,49] (Figure S1).

In sediment samples, the results ranged from 2.2 to 35.1 ng g^−1^ for two-ring PAHs (Nap), from 30.2 to 137.2 ng g^−1^ for three-ring PAHs (Acy, Ace, Flu, Phe, and Ant), from 59.2 to 241.2 ng g^−1^ for four-ring PAHs (Fla, Pyr, BaA, and Chr), from 191.3 to 490.3 ng g^−1^ for five-ring PAHs (BbF, BkF, BaP, and DahA), and from 11.2 to 109.4 ng g^−1^ for six-ring PAHs (BghiP and IcdP) (Table S8). In terms of individual PAHs in sediment, the composition characteristics were different from those in SPM and water (Figure S1). In sediment samples, the results showed the prevalence of four- and five-ring PAHs at most sites sampled, accounting for 36% and 42% of ΣPAHs in sediments, respectively. Liu et al. [50] reported a similar distribution of PAHs between water and sediment, confirming that HMW PAHs were mainly absorbed by sediment. This difference in distribution between water and sediment may be due not only to water solubility but also to bacterial degradation. In fact, the water solubility of PAHs probably decreases as the number of attached benzene rings increases, suggesting that HMW PAHs are less easily mobilized from solid substrates and dissolved into aquatic media than LMW PAHs and, as a result, they are less receptive to biodegradation. Instead, LMW PAHs have high solubility in water and greater benthic recycling, and were, therefore, more concentrated in the dissolved phase [51,52,53].

Such differences in pollutant composition between individual PAHs may be caused by different input methods and characteristics of PAHs. Firstly, river water receives direct PAH inputs from various sources, including wastewater discharge, runoff, atmospheric fallout, and so on. Secondly, low-molecular-mass PAHs gradually decrease as a result of degradation and adsorption, and only those PAHs that have relatively high molecular mass and are more resistant to degradation can resist such pressures to reach the sediment bed. Thirdly, water conditions, which change with the seasons, change the state of the water column process by mechanisms including dissolution, adsorption, desorption, degradation, and deposition [2,54,55].

**Figure 2:**
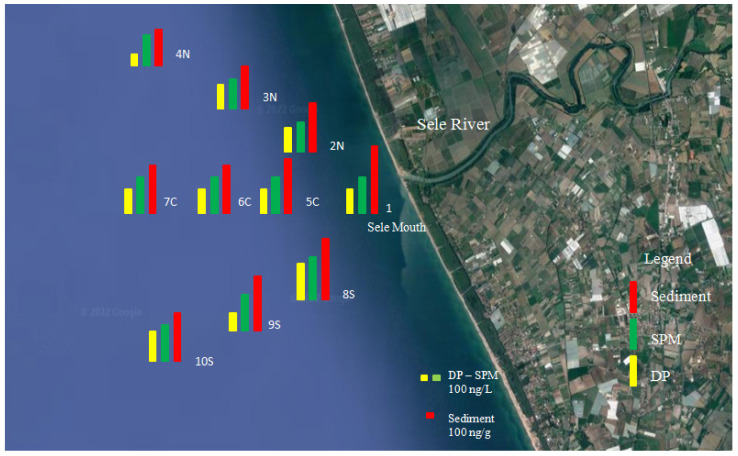
Spatial and temporal distributions of PAHs in the water-dissolved phase (DP, ng L^−1^), suspended particulate matter (SPM, ng L^−1^), and sediment (ng g^−1^ dry wt) of the Sele River and estuary, southern Italy.

**Figure 3:**
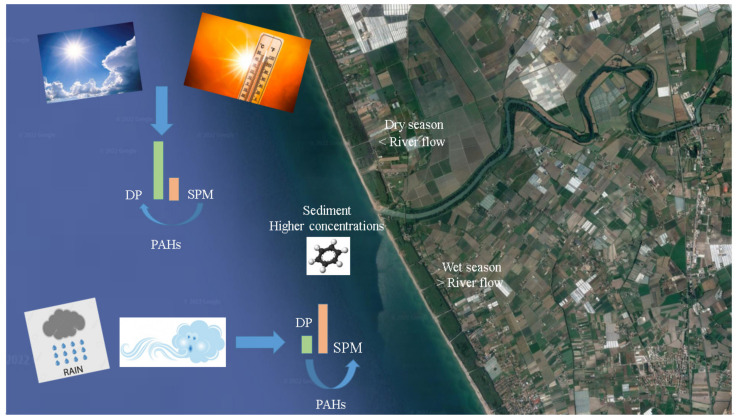
PAH distribution in water, suspended particulate matter, and sediment.

Spatial distribution data showed that concentrations reached their peak values at sites near the river mouth, while the levels of PAHs at other sites decreased from location one (river mouth) to four (1500 mt). In the Tyrrhenian Sea, PAH concentrations ranged in general from high values near the river outflow to low values in offshore areas (Figure 2). At 500 mt of river outflow, the PAH concentrations were close to those at the Sele mouth (Figure 2). The concentrations at the sampling sites then decreased at 1000mt from the river outflow and more still at 1500 mt. From the Sele mouth, the PAH load moved into the Tyrrhenian Sea southward (Figure 2). As can be seen from the results obtained, the trend in the concentrations indicated a decrease from the mouth towards 1500 mt at sea. This may depend both on the flow of the river, which varies according to the season, and on the diluting effect of the sea. The seasonal variation in PAH concentrations depends on the hydrological conditions, which may cause dilution ratio variations [56,57]. Therefore, a high river flow rate resulted in a higher dilution ratio during the wet season floods and caused a decrease in the PAH concentrations in both the Sele River and its estuary. In the area of the sea where the sampling sites are located, the flow direction of the seawater moves south. The marine flow influences the concentrations and the distribution of the PAHs, which also change according to the seasons. This is influenced by the currents and characteristic winds of the Mediterranean Sea [58]. When the flow of the sea changes, several factors change that can contribute to altering the concentrations of the studied compounds: temperature, salinity, and often also “color” (more or less cloudy) [59]. These factors lead to changes in the density of the water. The occurrence of flow events implies a high presence of suspended solid matter of terrestrial origin, as well the resuspension of sediments caused by turbulence and the transport of the associated PAHs downstream. In contrast, when low-flow conditions predominate and previous flood events are long past, the settling of suspended matter and the associated storage of PAH particles in the sediment are favored [60]. The PAH sources present in the study area that may contain seawater are represented by the various industries present in the area and agricultural activities as well. The results showed that the PAH concentrations in DP decreased from July to February, in parallel with the increase in rainfall, which could cause dilution ratio variations. Therefore, the decrease in PAH concentrations moving from the Sele River mouth to the Mediterranean Sea was also affected by the high flow in the rainfall season, which resulted in an even higher dilution ratio. The lowest concentrations in SPM were recorded in the dry season (July) due to the decrease in flow and the greater stagnation of SPM, which led PAHs with a greater polarity to shift from SPM to DP (Figure 3).

Based on these results, it can be concluded that the loads and migrations of PAHs between different phases at each sampling site of the Sele River were related to variations in the flow during rainy and dry seasons. Therefore, a high concentration of PAHs in sediments indicated that the contamination of PAHs in the Sele River and its estuary might be caused by the historical input of PAHs. The total load of PAHs that flowed into the Tyrrhenian Sea was evaluated to estimate the input of PAHs drained from rainwater outflow, tributary inflow, wastewater treatment plants, industrial effluent discharge, agricultural runoff, atmospheric deposition, and dredged material disposal. The total PAH loads contributed to the Tyrrhenian Sea from the Sele River were calculated considering the concentration values of the individual PAHs at the river mouth in the four months of sampling. The mean of the total concentrations was then multiplied by the annual average flow rate (m^3^/year) of the Sele River. The load was calculated as about 1807.9 kg/year.

### 3.2. PAH Fugacity in the Aquatic System

Since water and sediment in aquatic ecosystems are subject to dynamic equilibration, it would be useful to identify the transport processes and fate of PAHs so that an estimation of the distribution between water and sediment could yield useful information. The values obtained in this study for the sediment-water equilibrium partitioning coefficient (log *K_oc_*), in situ sediment-water distribution coefficient (log *K_oc_′*), and fugacity fraction (ff) of PAHs at the 10 sampling sites are shown in Table 2.

The mean values of log *K′_oc_* ranged from 0.41 to 5.99, but the average log *K′_oc_* values for PAH compounds, except two-three-ring PAHs, were lower than their corresponding log *K_oc_*. This indicates that these compounds were saturated in the water-dissolved phase, and their net flux was from the water into sediment. Overall, the difference between in situ log *K′_oc_* and the corresponding log *K_oc_* indicated non-steady-state conditions for the PAHs in the water-sediment system, but the differences between the log *K′_oc_* and log *K_oc_* values of HMW PAHs were relatively large, suggesting that the non-steady-state increased for HMW PAHs. In fact, the difference between log *K_oc_′* and the corresponding log *K_oc_* indicates the equilibrium state of PAHs in the aquatic system [28]. If the average log *K_oc_′* is lower than the corresponding Koc and Kow, PAHs are more absorbed into the sediment phase than exchanged into the water phase [27]. Therefore, LMW PAHs were usually dominant in water, and they tend to be released from SPM to water; HMW PAHs were prevalent in sediment, and they tend to be adsorbed onto SPM from water [28,62].

The fugacity fraction ff was used to evaluate the equilibrium status of the organic pollutants and to better understand the interactions between the phases [27,63]. In the sediment-water of the Sele River, the ff values of the 16 PAHs were 0.04–0.28, i.e., lower than 0.3, causing a net flux of these PAHs from the water into the sediment.

### 3.3. Risk Assessment of PAHs

Several evaluation tools, such as sediment quality guidelines (SQGs) and the toxic equivalent quotient (TEQ), are frequently used for preliminary analysis and evaluation of the ecological risk faced by aquatic environments. These methods can rapidly and effectively evaluate the potential risk level to aquatic organisms induced by contaminant concentrations in the environmental medium.

In the Sele River, the obtained data showed concentrations of PAHs lower than the PEL and ERM values; however, for TEL and ERL values, not all concentrations were lower (Table 3). Moreover, the seasonal differences also influenced the risk assessment, which was higher for the compounds analyzed (DP and SPM) in July and lower in February, in relation to the concentrations found.

For individual compounds, TEL values were higher for Acy, Ace, and DahA for all samples; for Nap and Flu in 20% of samples; and for Bap in 70% of samples, indicating that adverse effects may occasionally exist. However, the mean concentrations of the detected PAHs were lower than their respective PEL values.

The amounts of individual PAHs did not exceed their respective ERM values, but the ERL values were exceeded for Ace in 50% of samples, for Flu in 20% of samples, and for DahA in all samples. The data obtained confirmed the presence of PAHs at some sites, showing that the environmental integrity of Sele River was at risk.

In this study, the total TEQ_PAHs_ ranged from 137.3 to 292.6 ngTEQ/g; the highest values were measured at the river mouth and site eight, while all other sampling sites presented TEQ_PAHs_ values under the safe level.

Qu et al. [14] evaluated the PAH levels in the sediment of the Gulfs of Naples and Salerno, reporting TEQ_PAHs_ values ranging from 0.07 to 1425 ngTEQ/g; Arienzo et al. [16] studied the PAH levels in the sediment of the Gulf of Pozzuoli, with values between 1580 and 501.70 ngTEQ/g.

### 3.4. Source Identification by PAH Diagnostic Ratios

**Figure 4:**
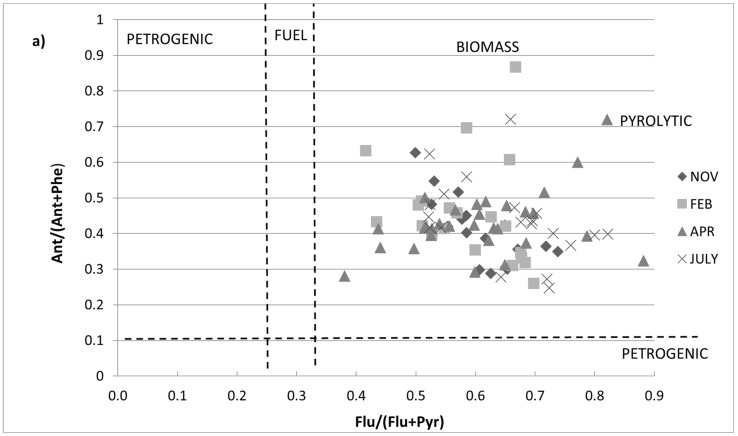

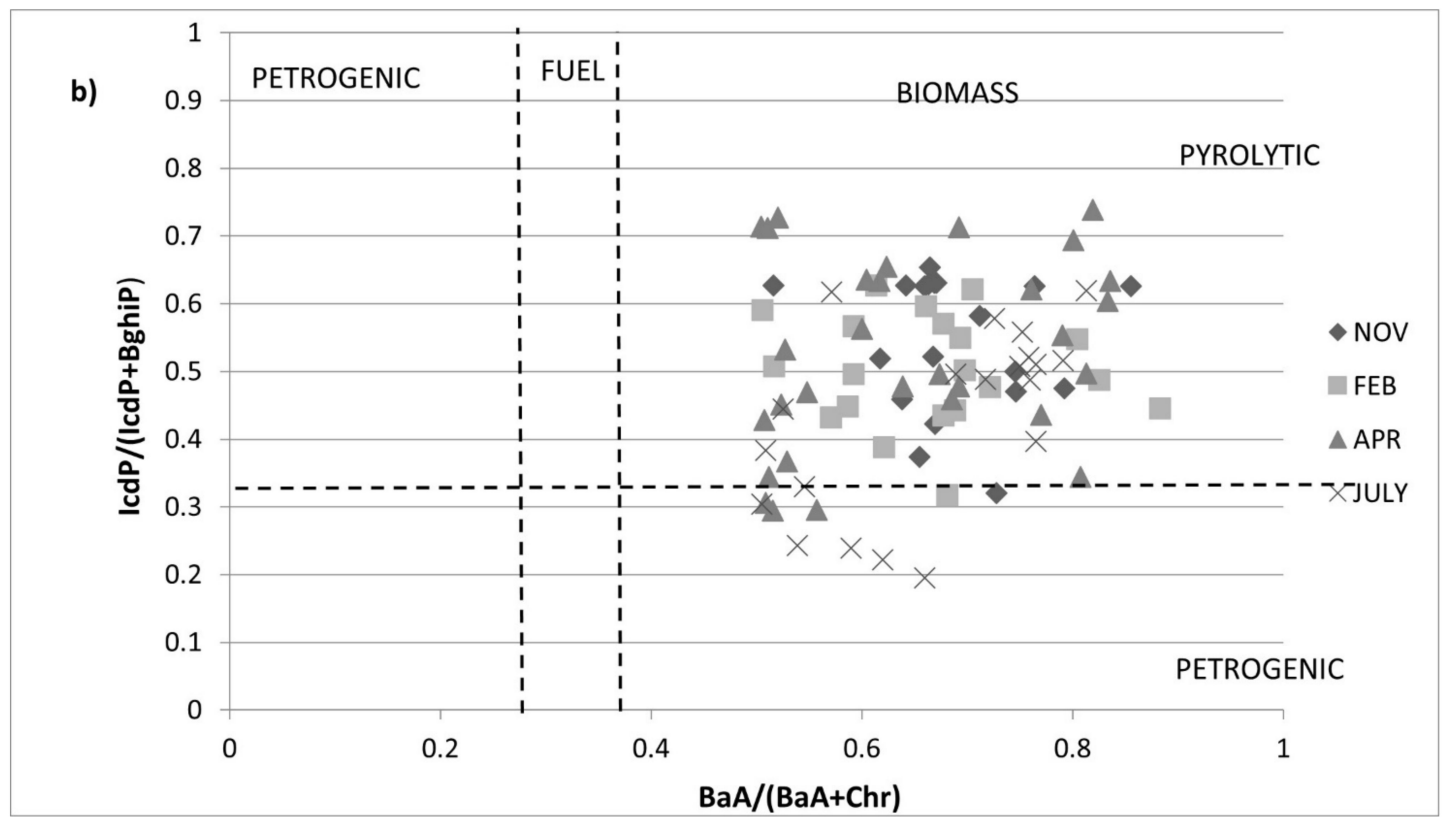
Cross plots of the values of (**a**) Flu/(Flu + Pyr) versus Ant/(Ant + Phe) and (**b**) BaA/(BaA + Chr) versus IcdP/(IcdP + BghiP) for all sample data from the Sele River and its estuary.

Data from this study highlighted a prevailing pattern of pyrolytic inputs of PAHs in the Sele River and its estuary. In effect, the results demonstrated that the Ant/(Ant + Phe) ratio was ˃0.1 in DP, SPM, and sediment (means of 0.40, 0.41, and 0.43, respectively), which assigned the origin of the PAHs to pyrogenic sources. Moreover, Flu/(Flu + Pyr) ratios allowed us to differentiate petroleum origins from combustion processes and make the distinction between such sources [66,67]. For Flu/(Flu + Pyr), low ratios (<0.40) indicate petroleum, intermediate ratios (0.40–0.50) indicate liquid fossil fuel combustion, and ratios >0.50 are characteristic of grass, wood, and coal combustion. In the Sele River and its estuary, a ratio value of Flu/(Flu + Pyr) ˃ 0.5 was found in the dissolved phase, particulate matter, and sediment, indicating that combustion was the main source of pollution there (Figure 4a).

Ratio values of BaA/(BaA + Chr) ˃ 0.35 and InP/(InP + BghiP) ˃ 0.35 were found in the dissolved phase, particulate matter, and sediment, indicating a mixed source of petroleum and combustion (Figure 4b). The ratio results from the samples indicated that they were mainly contaminated by combustion. In general, atmospheric particles emitted from factories may be transported and deposited into the river. Moreover, industrial wastewater and vehicle emissions also suggest a pyrolytic origin for PAH pollution in the area. Among the pollutants evaluated in this study, Per was probably the most important diagenetic PAH found; therefore, the high concentration of this compound compared to the others could indicate a natural origin [55,68,69,70].

In fact, it has been indicated that amounts of Per above 10% of the total penta-aromatic isomers suggest a probable diagenetic input, whereas those samples in which Per accounts for less than 10% suggest a probable pyrolytic origin of the compound. In this study, the amount of Per detected in all sediment samples was very low (range 1.97–9.72 ng g^−1^) and contributed less than 2% to the penta-aromatic isomers, indicating a pyrolytic origin of these pollutants. Differences in PAH spatial distributions in different periods are expected to be due to different sources of PAH inputs, water conditions, and the characteristics of individual PAHs. In the dry period, the river is stagnant, which weakens the transport of the pollutants from upstream to downstream, and the higher values at some sites may be the result of some highly local inputs. Special PAH ratios such as BaA/(BaA + Chr) and IcdP/(IcdP + BhiP) indicated that, in July, in dry season weather conditions, the PAHs found in the Sele River were primarily from petrogenic sources, while under wet weather season conditions, they were from pyrolytic sources.

**Figure 5:**
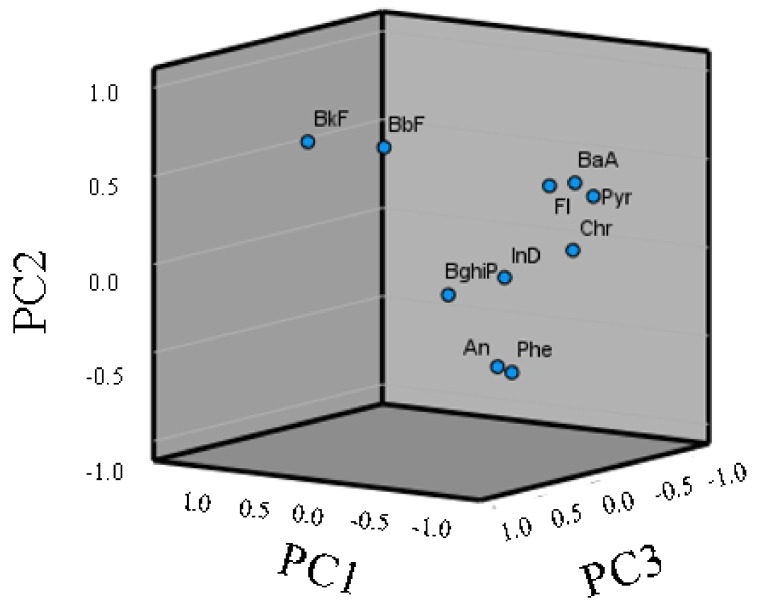
Principal component analysis (PCA) of PAH composition in samples from the Sele River estuary, southern Italy.

PCA was used to quantitatively assess PAH origins, and molecular ratios between isomers were used: PAHs were represented by three PC factors (PC1, PC2, and PC3), and the extracted eigenvectors showed 47% for PC1 (Figure 5). This factor was mostly loaded by the four-ring PAHs Pyr, Flu, Chr, and BaA and the six-ring PAHs IcdP and BghiP. PAHs such as Pyr and Chr are indicators for coal burning, while Flu may indicate combustion. Similar behavior was observed for the ratio IcdP/(IcdP + BghiP, which indicates a mixed source of petroleum and combustion [71]. PC2 (21%) represented HMWs belonging to the five-ring PAHs BbF and BkF. High-molecular-weight PAHs such as these indicate pyrolysis and incomplete biomass combustion [72]. PC3, in contrast, contributed only 13% of the total load and represented the three-ring PAHs Phe and Ant (Figure 5). Therefore, the LMW/HMW ratio was low (<1 for each site), which is an indication of a pyrolytic origin of PAHs at these sites [73]. The PCA and diagnostic ratios indicate that the origins of contamination by PAHs in the Sele River were due to pyrolytic sources and combustion sources, such as gasoline burning and fuel and coal burning.

## 4. Conclusions

This paper offers important data on PAH concentrations and composition in the Sele River where it empties into the Tyrrhenian Sea (Central Mediterranean Sea), southern Italy, and presents the first comprehensive study of PAHs in water, SPM, and sediment in that area. The levels of LMW PAHs were particularly high in water samples, while the levels of HMW PAHs were predominant in sediment samples. A determination of the diagnostic ratio of PAHs revealed that the main PAH sources were pyrolytic and suggested that the majority of this pollution derived from vehicle traffic and combustion processes. The exchange of PAHs between water and sediment occurs in the direction of adsorption into the sediment from water. Regarding the risk assessment, the concentrations of many single PAHs at a number of sites were above ERL and/or TEL (and below ERM and/or PEL), which would on occasion yield negative environmental consequences. However, the toxic equivalent concentration (TEQ) of carcinogenic PAHs suggests that the Sele River basin presents a definite carcinogenic risk. Thus, the waters of the Sele River should be continuously monitored, as PAHs could lead to negative consequences for its aquatic ecosystems and organisms.

## Figures and Tables

**Table 1:** Description of the sampling sites and concentrations of PAHs in the water-dissolved phase (DP), suspended particulate matter (SPM), and sediment of the Sele River, southern Italy. (The values in brackets represent the values of PAH concentrations in SPM expressed in ng g^−1^ dry wt, after drying the filters in an air-heated oven and weighing them).

Sampling Location			ΣPAHs								
Site Number Identification	Site Characteristics	Site Location	Dissolved Phase (ng L^−1^)				Particulate Phase (ng L^−1^) (ng g^−1^ Dry wt)				Sediment (ng g^−1^ Dry wt)
			Apr	Jul	Nov	Feb	Apr	Jul	Nov	Feb	Apr
1 (river water)	Sele river source	40°28′55″ N 14°56′33″ E	419.3	567.2	487.3	309.9	520.1 (41,364.1)	276.1 (28,122.3)	234.8 (19,865.6)	654.3 (23,487.2)	871.1
2 (sea water)	River mouth at 500 mt north	40°29′04″ N 14°56′14″ E	204.2	387.3	471.2	200.0	332.3 (30,542.6)	144.8 (18,657.1)	138.3 (6068.5)	381.0 (26,589.1)	712.4
3 (sea water)	River mouth at 500 mt central	40°29′12″ N 14°55′56″ E	226.5	552.3	408.2	331.8	233.9 (74,510.7)	182.3 (26,789.8)	128.3 (58,745.8)	277.7 (16,895.9)	724.3
4 (sea water)	River mouth at 500 mt south	40°29′20″ N 14°55′38″ E	487.3	560.2	509.1	334.1	504.2 (41,263.6)	261.2 (48,756.3)	181.2 (5986.8)	507.2 (47,596.2)	852.2
5 (sea water)	River mouth at 1000 mt north	40°28′55″ N 14°56′12″ E	309.5	497.3	424.4	121.9	370.3 (29,865.2)	125.1 (11,587.3)	204.5 (13,501.6)	589.9 (2843.2)	649.5
6 (sea water)	River mouth at 1000 mt central	40°28′55″ N 14°55′50″ E	227.3	498.3	529.3	249.7	328.7 (10,859.8)	214.7 (65,741.0)	190.2 (18,459.2)	461.1 (14,896.2)	708.1
7 (sea water)	River mouth at 1000 mt south	40°28′55″ N 14°55′28″ E	302.1	499.2	502.6	262.3	467.6 (36,587.2)	294.9 (24,189.2)	188.2 (10,453.2)	369.1 (4875.2)	744.3
8 (sea water)	River mouth at 1500 mt north	40°28′47″ N 14°56′16″ E	300.2	112.3	289.7	10.1	367.9 (19,845.5)	121.9 (10,354.3)	219.0 (16,181.1)	192.3 (5489.5)	331.7
9 (sea water)	River mouth at 1500 mt central	40°28′39″ N 14°55′56″ E	361.7	331.2	424.8	175.9	482.1 (86,412.3)	240.2 (66,587.4)	185.7 (58,476.5)	277.9 (13,489.2)	602.1
10 (sea water)	River mouth at 1500 mt south	40°28′30″ N 14°55′38″ E	471.0	489.3	509.1	207.1	545.8 (85,647.1)	387.3 (29,875.1)	173.2 (39,485.2)	451.7 (8746.2)	683.2

**Table 2:** Comparison of log *K_oc_* and log *K′_oc_* for polycyclic aromatic hydrocarbons (PAHs) at the water-sediment interface and the fugacity fraction (*ff*) in the study area.

PAHs	log *K_oc_* ^a^	log *K′_oc_* (Mean)	*ff*
Nap	3.11	3.25	0.05
Any	3.51	3.78	0.10
Ace	3.43	4.15	0.06
Flu	3.70	3.58	0.04
Phe	3.87	4.22	0.06
Ant	3.40	4.00	0.06
Fla	3.70	4.79	0.09
Pyr	4.66	3.88	0.08
BaA	5.30	4.29	0.12
Chr	5.43	4.05	0.18
Bbf	5.36	1.21	0.27
Bkf	5.57	1.18	0.23
BaP	5.61	2.22	0.12
IcdP	6.64	0.41	0.28
DahA	6.22	2.10	0.10
Bghip	6.90	0.83	0.05

^a^ Guo et al. [61].

**Table 3:** A comparison of the TEL, PEL, ERL, and ERM guideline values (µg Kg^−1^) for polycyclic aromatic hydrocarbons and the data found for the Sele River, southern Italy.

	PAHs																
	Nap	Acy	Ace	Flu	Phe	Ant	Fla	Pyr	BaA	Chr	BbF	BkF	BaP	DahA	BghiP	IcdP	∑PAHs
TEL ^a^	34.6	5.87	6.71	21.2	86.7	46.9	113	153	74.8	108	-	-	88.8	6.22	-	-	1684
Percentage of samples over the TEL	20	100	100	40	0	0	0	0	0	0			70	100			0
PEL ^a^	391	128	88.9	144	544	245	1494	1398	693	846	-	-	763	135	-	-	16770
Percentage of samples over the PEL	0	0	0	0	0	0	0	0	0	0			0	10			0
ERL ^b^	160	44	16	19	240	85	600	665	261	384	-	-	430	63.4	-	-	4022
Percentage of samples over the ERL	0	0	50	20	0	0	0	0	0	0			0	100			0
ERM ^b^	2100	640	500	540	1500	1100	5100	2600	1600	2800	-	-	1600	260	-	-	44792
Percentage of samples over the ERM	0	0	0	0	0	0	0	0	0	0			0	0			0

^a^ Long et al. [64]. ^b^ MacDonald et al. [65].

## Data Availability

The datasets obtained and analyzed in the current study are available from the corresponding author on reasonable request.

## Supplementary Materials

The following are available online at https://www.mdpi.com/article/10.3390/toxics10070401/s1, Table S1: Characteristic Ions of the analyzed PAHs; Table S2: Individual PAHs recovery values; Table S3: Description of concentration of PAHs in sediment samples of the Sele River, southern Italy; Table S4: Description of concentration of PAHs in water dissolved phase (DP) samples of the Sele River, southern Italy; Table S5: Description of concentration of PAHs in suspended particulate matter (SPM) samples of the Sele River, southern Italy; Table S6: Parameter of GC-MS system; Table S7: Validation parameter values of PAHs in water samples and SPM samples; Table S8: Validation parameter values of PAHs in Sediment samples; Figure S1: Chromatograms obtained in different phase of Sele River. (a) PAHs chromatogram identified in a water sample (Dissolved phase) of Sele River. (b) PAHs chromatogram identified in a Suspended particulate matter (SPM) sample of Sele River. (c) PAHs chromatogram identified in a water sample (Dissolved phase) of Sele River. (d) PAHs chromatogram identified in a Suspended particulate matter (SPM) sample of Sele River. (e) PAHs chromatogram identified in Sediment sample of Sele River. (f) PAHs chromatogram identified in Sediment sample of Sele River.
