# Supplementary material for: Polycyclic Aromatic Hydrocarbons (PAHs) in the Dissolved Phase, Particulate Matter, and Sediment of the Sele River, Southern Italy: A Focus on Distribution, Risk Assessment, and Sources
Source: Toxics. 2022 Jul 19;10(7):401. doi: 10.3390/toxics10070401 (PMC9324633; doi:10.3390/toxics10070401)
Supplement: Supplementary file 1 [file toxics-10-00401-s001.zip › toxics-1792423-supplementary.pdf]

Polycyclic Aromatic Hydrocarbons (PAHs) in the Dissolved Phase, Particulate Matter, and Sediment of the Sele River, Southern Italy: A Focus on Distribution, Risk Assessment, and Sources

Paolo Montuori [1,*], Elvira De Rosa [1], Fabiana Di Duca [1], Bruna De Simone [1], Stefano Scippa [1], Immacolata Russo [1], Pasquale Sarnacchiaro [2] and Maria Triassi [1]

Table S1. Individual PAHs recovery values

PAHs	Recovery %
Naphthalene	88
Acenaphthylene	82
Acenaphthene	89
Fluorine	80
Anthracene	86
Phenanthrene	84
Fluoranthene	80
Pyrene	80
Benz(a)anthracene	88
Chrysene	80
Benzo(b)fluoranthene	94
Benzo(k)fluoranthene	94
Benzo(a)pyrene	97
Indeno(1,2,3-cd)pyrene	89
Dibenz(a,h) anthracene	91
Benzo(g,h,i) perylene	96
Benzo(a)pyrene$_{d12}$	94
Indeno(1,2,3-cd)pyrene $_{d12}$	91

Table S2. Characteristic Ions of the analyzed PAHs

Compound	Primary Ion	Secondary Ion
Naphthalene	128.10	64.00
Acenaphthylene	152.10	76.10
Acenaphthene	154.20	-
Fluorine	166.10	164.10
Anthracene	178.10	89.10
Phenanthrene	178.10	89.10
Fluoranthene	202.10	101.10
Pyrene	202.10	101.10
Benz(a)anthracene	228.10	114.10
Chrysene	228.10	114.10
Benzo(b)fluoranthene	252.20	126.20
Benzo(k)fluoranthene	252.20	126.20
Benzo(a)pyrene	252.10	126.10
Indeno(1,2,3-cd)pyrene	276.20	138.10
Dibenz(a,h) anthracene	278.20	139.20
Benzo(g,h,i) perylene	276.20	138.10

Table S3. Parameter of GC-MS system

Parameter	Set Condition
Gas Chromatografy (GC)	TRACE ™ 1310
Injection volume	1 µL
Inlet temperature	300 °C
Capillary column	TG-5MS (30 mm ×0.25 mm inner diameter × 0.25 µm film)
Injection mode	Splitless
Carrier gas	Helium, 1mL/min
Mass selective detector (MS)	ISQ™ 7000 Single Quadrupole Mass
Ionization Mode	Electron Ionization (EI)
Transfer Line Temperature	280°C
Ion source Temperature	230°C
Electronic energy	70 eV
Scan mode	Selective Ion Monitoring (SIM)

Table S4. Validation parameter values of PAHs in water samples and SPM samples

PAHs Compound	Linearity (R^2)	LOD (ppb)	LOQ (ppb)
Naphthalene	0.993	1.57	5.25
Acenaphthylene	0.998	1.50	5.01
Acenaphthene	0.995	1.53	5.10
Fluorine	0.994	1.46	4.87
Anthracene	0.992	1.53	5.11
Phenanthrene	0.993	1.55	5.30
Fluoranthene	0.994	1.31	5.20
Pyrene	0.996	1.48	4.90
Benz(a)anthracene	0.997	1.52	4.80
Chrysene	0.995	1.54	5.10
Benzo(b)fluoranthene	0.996	1.44	5.00
Benzo(k)fluoranthene	0.998	1.50	5.11
Benzo(a)pyrene	0.993	1.59	5.30
Indeno(1,2,3-cd)pyrene	0.999	1.31	4.81
Dibenz(a,h) anthracene	0.999	1.42	5.31
Benzo(g,h,i) perylene	0.992	1.62	5.40

Table S5. Validation parameter values of PAHs in Sediment samples

PAHs Compound	Linearity (R^2)	LOD (ppb)	LOQ (ppb)
Naphthalene	0.993	1.63	5.44
Acenaphthylene	0.998	1.90	6.30
Acenaphthene	0.995	1.55	5.17
Fluorine	0.994	1.86	6.23
Anthracene	0.992	1.56	5.21
Phenanthrene	0.993	1.59	5.30
Fluoranthene	0.994	1.65	5.50
Pyrene	0.996	1.83	6.10
Benz(a)anthracene	0.997	1.56	5.20
Chrysene	0.995	1.80	6.0
Benzo(b)fluoranthene	0.996	1.62	5.41
Benzo(k)fluoranthene	0.998	1.61	5.39
Benzo(a)pyrene	0.993	1.64	5.48

Indeno(1,2,3-cd)pyrene	0.999	1.82	6.08
Dibenz(a,h) anthracene	0.999	1.71	5.71
Benzo(g,h,i) perylene	0.992	1.70	5.68

Table S6. Description of concentration of PAHs in water dissolved phase (DP) samples of the Sele River, southern Italy.

	Nap	Acy	Ace	Flu	Phe	An	Fl	Pyr	BaA	Chr	BbF	BkF	BaP	DahA	BghiP	InP
**November**																
River Mouth (sea water)	89,29	44,15	34,57	39,76	46,57	54,76	35,66	39,88	20,45	12,69	12,74	10,90	21,90	5,18	10,45	8,38
Nord estuary at 500mt (sea water)	80,45	25,63	32,88	31,77	45,69	53,15	74,68	37,36	12,76	6,27	5,65	8,24	36,87	1,49	6,75	11,52
Nord estuary at 1000mt (sea water)	45,12	12,10	26,49	44,87	90,11	38,70	65,38	34,73	6,64	3,29	2,38	5,14	28,82	1,57	7,03	12,01
Nord estuary at 1500mt (sea water)	22,25	12,71	19,79	16,84	77,44	34,10	32,48	17,44	8,47	1,43	2,62	3,96	18,29	1,80	7,54	12,62
Central estuary at 500mt (sea water)	80,41	14,65	24,98	32,98	37,19	75,88	25,90	34,99	17,86	9,06	7,87	9,33	14,77	2,34	7,43	12,58
Central estuary at 1000mt (sea water)	69,42	12,20	46,85	52,86	103,57	42,00	75,93	45,43	11,10	3,86	2,48	7,77	34,64	1,69	7,28	12,26
Central estuary at 1500mt (sea water)	46,53	12,04	32,75	33,84	92,01	49,41	63,10	22,38	9,99	3,09	2,38	5,80	30,91	1,51	7,14	11,95
South estuary at 500mt (sea water)	95,09	25,22	56,39	48,75	37,57	45,77	37,49	36,88	31,38	29,41	9,05	8,58	21,79	8,65	7,33	9,88
South estuary at 1000mt (sea water)	56,10	12,46	52,14	45,77	55,63	62,61	79,87	41,35	11,48	7,34	6,25	7,23	44,29	2,44	6,58	11,08
South estuary at 1500mt (sea water)	61,13	12,59	38,27	52,32	93,23	53,31	75,36	29,59	9,49	4,90	4,72	6,17	46,89	1,55	7,32	12,26
**February**																
River Mouth (sea water)	40,62	13,79	14,47	19,09	6,99	13,50	23,87	19,04	34,68	9,11	7,15	16,78	24,70	8,56	24,98	32,65
Nord estuary at 500mt (sea water)	18,02	13,14	10,63	19,41	11,01	13,74	20,98	10,93	15,61	7,42	5,05	7,62	13,63	5,49	11,74	15,58
Nord estuary at 1000mt (sea water)	11,06	12,02	4,06	16,25	3,56	5,74	7,42	5,26	9,47	4,86	3,31	5,37	9,58	3,80	8,14	12,02
Nord estuary at 1500mt (sea water)	2,46	0,24	0,85	0,35	0,29	0,66	0,87	0,47	1,00	0,77	0,88	0,99	0,18	0,10	0,01	0,10
Central estuary at 500mt (sea water)	29,08	11,85	20,03	31,11	38,70	26,43	41,68	22,41	16,78	7,98	6,31	7,16	36,12	6,43	16,83	12,95
Central estuary at 1000mt (sea water)	19,11	12,26	15,18	20,56	29,29	12,97	29,04	13,48	9,48	4,11	5,54	4,88	33,41	12,01	14,15	14,27
Central estuary at 1500mt (sea water)	7,58	11,96	7,31	11,67	15,99	8,22	15,53	10,38	8,36	3,50	3,47	4,43	28,45	8,70	11,52	18,89
South estuary at 500mt (sea water)	34,40	15,39	28,40	30,37	38,91	28,50	17,39	10,04	11,48	11,24	9,63	7,57	41,03	9,54	16,46	23,76
South estuary at 1000mt (sea water)	20,46	12,06	21,79	19,30	23,24	18,52	30,14	18,02	8,50	7,95	7,65	7,61	30,81	7,61	14,15	14,59
South estuary at 1500mt (sea water)	11,93	11,33	16,02	16,96	11,70	18,24	24,14	16,36	8,09	5,57	6,56	5,23	28,67	2,83	11,84	11,67
**April**																
River Mouth (sea water)	79,26	33,64	32,98	31,75	40,87	39,98	40,76	30,98	20,22	13,46	14,29	10,55	8,99	5,95	9,31	6,35
Nord estuary at 500mt (sea water)	65,46	12,36	18,49	11,47	10,48	13,57	14,36	10,48	9,36	4,51	4,30	7,46	6,47	4,83	5,37	5,28
Nord estuary at 1000mt (sea water)	63,37	29,27	37,47	25,47	21,34	19,37	23,58	23,47	8,56	3,66	10,03	6,49	5,68	8,58	7,29	15,87
Nord estuary at 1500mt (sea water)	52,33	25,69	29,25	27,13	20,58	26,63	34,94	23,41	10,71	2,56	4,25	9,97	14,25	2,59	6,55	9,38
Central estuary at 500mt (sea water)	40,26	14,86	20,60	11,25	19,16	16,59	20,59	11,47	16,59	13,36	7,25	8,88	6,57	6,41	5,90	6,86

Central estuary at 1000mt (sea water)	38,26	14,86	20,60	11,25	19,16	16,59	24,03	12,47	17,59	14,36	8,25	7,88	7,27	7,41	7,90	4,86
Central estuary at 1500mt (sea water)	42,06	22,03	24,03	25,03	27,26	15,26	19,03	16,03	15,20	18,98	7,06	6,99	6,25	5,05	6,56	4,99
South estuary at 500mt (sea water)	85,29	42,15	36,57	37,76	44,57	55,76	35,66	40,88	20,45	11,69	10,74	10,90	21,90	6,18	9,45	8,38
South estuary at 1000mt (sea water)	62,62	13,79	14,47	19,09	26,99	13,50	20,87	19,04	14,68	7,11	5,15	16,78	16,70	13,56	24,98	20,25
South estuary at 1500mt (sea water)	58,26	25,63	32,88	31,77	45,69	53,15	74,68	37,36	25,76	10,27	5,65	8,24	36,87	1,49	12,75	11,52
**July**																
River Mouth (sea water)	87,03	69,55	55,02	58,03	46,25	49,05	50,02	41,25	19,42	14,81	16,88	13,62	11,26	9,34	14,26	11,42
Nord estuary at 500mt (sea water)	69,05	36,27	31,25	36,26	38,16	48,26	34,26	31,18	12,50	11,18	9,48	9,91	8,29	6,30	8,03	7,88
Nord estuary at 1000mt (sea water)	89,29	44,15	44,57	40,76	46,57	54,76	35,66	39,88	20,45	12,69	12,74	10,90	11,90	11,18	12,45	9,38
Nord estuary at 1500mt (sea water)	19,07	11,06	7,88	7,60	8,58	9,47	9,75	7,90	9,88	3,43	2,99	1,29	3,88	2,39	4,19	3,06
Central estuary at 500mt (sea water)	80,03	65,55	52,02	60,03	44,25	45,05	50,02	40,25	30,42	20,81	15,88	10,62	10,26	9,34	11,26	6,52
Central estuary at 1000mt (sea water)	79,85	42,15	46,57	47,76	44,57	55,76	35,66	40,88	20,45	16,69	10,74	10,90	21,90	6,18	9,25	9,08
Central estuary at 1500mt (sea water)	70,06	22,03	24,03	25,03	27,26	30,26	29,03	23,03	21,20	18,98	7,06	6,99	7,25	5,05	6,56	7,49
South estuary at 500mt (sea water)	80,03	69,55	55,02	58,03	46,25	49,05	50,02	41,25	19,42	14,81	16,88	13,62	11,26	9,34	14,26	11,42
South estuary at 1000mt (sea water)	85,29	44,15	37,57	41,76	46,57	50,76	41,66	40,88	22,45	12,69	12,74	12,90	21,90	6,18	13,25	8,48
South estuary at 1500mt (sea water)	80,29	42,15	36,57	37,76	45,57	55,76	41,66	40,88	21,45	11,69	11,74	12,90	21,90	7,18	13,45	8,38

Table S7. Description of concentration of PAHs in suspended particulate matter (SPM) samples of the Sele River, southern Italy.

	Nap	Acy	Ace	Flu	Phe	An	Fl	Pyr	BaA	Chr	BbF	BkF	BaP	DahA	BghiP	InP
**November**																
River Mouth (sea water)	10,24	11,48	15,20	21,95	10,52	13,47	27,80	14,55	10,71	3,65	14,29	8,49	31,43	15,36	12,84	12,84
Nord estuary at 500mt (sea water)	9,88	7,26	8,57	9,97	8,75	6,85	7,58	10,73	8,42	8,80	11,79	6,72	10,69	6,63	8,58	7,26
Nord estuary at 1000mt (sea water)	10,47	13,76	12,43	17,75	14,25	10,55	10,59	9,36	15,46	8,76	16,24	11,15	14,21	10,16	15,26	14,16
Nord estuary at 1500mt (sea water)	12,37	10,26	8,57	9,97	15,75	13,85	17,58	15,73	10,42	15,80	11,79	12,72	17,69	16,63	18,58	12,26
Central estuary at 500mt (sea water)	5,88	6,26	8,57	7,97	8,75	6,85	7,58	10,73	8,42	8,80	11,79	6,72	10,69	5,63	8,58	5,16
Central estuary at 1000mt (sea water)	13,21	12,77	9,15	22,33	11,27	12,03	15,10	11,34	19,65	6,68	5,05	7,33	17,31	5,10	11,23	11,17
Central estuary at 1500mt (sea water)	12,26	11,49	12,63	18,41	11,54	10,74	13,61	12,25	14,03	6,42	12,05	7,02	12,26	7,01	11,27	12,24
South estuary at 500mt (sea water)	10,21	9,77	9,15	22,33	11,27	12,03	15,10	11,34	19,65	6,68	5,05	7,33	10,01	6,10	14,03	11,17
South estuary at 1000mt (sea water)	15,92	11,17	12,57	22,63	10,10	9,65	9,88	9,47	18,22	7,38	5,55	8,44	14,75	7,22	10,26	15,01
South estuary at 1500mt (sea water)	11,21	11,68	10,83	16,66	13,19	9,56	12,78	10,30	16,04	5,99	4,08	6,31	15,66	7,26	14,71	6,93
**February**																
River Mouth (sea water)	37,01	34,09	32,78	43,16	35,12	39,59	32,93	30,25	32,25	37,05	42,36	53,03	52,15	57,02	47,03	49,02
Nord estuary at 500mt (sea water)	34,46	14,78	31,43	25,05	24,03	27,06	25,06	25,37	16,69	11,76	14,26	11,03	31,05	32,03	29,06	28,03

Nord estuary at 1000mt (sea water)	20,59	12,34	16,93	20,51	26,24	13,03	30,54	14,55	11,42	4,41	5,14	7,81	38,61	14,09	23,15	18,39
Nord estuary at 1500mt (sea water)	11,33	11,24	12,52	20,36	22,81	7,26	24,42	10,58	10,50	2,04	2,79	6,05	13,63	11,30	12,39	13,12
Central estuary at 500mt (sea water)	20,77	23,02	26,36	33,57	30,26	38,36	34,87	40,26	32,07	21,06	50,59	52,03	51,03	45,15	42,58	47,96
Central estuary at 1000mt (sea water)	23,02	15,71	37,22	34,79	35,26	23,46	26,93	24,19	17,94	10,94	32,05	32,02	40,83	28,20	39,55	39,03
Central estuary at 1500mt (sea water)	16,30	11,58	17,48	20,85	28,70	12,92	29,09	14,85	19,78	4,80	5,45	38,05	33,08	43,03	37,17	36,02
South estuary at 500mt (sea water)	22,72	17,38	33,24	33,46	30,61	28,27	32,47	31,97	33,58	27,03	35,06	38,06	46,65	35,08	34,02	28,03
South estuary at 1000mt (sea water)	26,27	12,58	34,89	32,91	32,79	23,96	25,54	24,47	16,85	12,69	42,04	43,02	48,21	28,02	21,45	26,05
South estuary at 1500mt (sea water)	15,72	12,70	18,79	20,61	27,92	14,82	31,61	15,17	25,48	3,38	5,39	9,13	30,30	15,66	14,50	16,73
**April**																
River Mouth (sea water)	30,59	14,82	28,87	32,82	33,60	29,35	36,03	37,47	35,47	36,47	32,74	31,47	43,13	36,48	32,26	28,47
Nord estuary at 500mt (sea water)	5,32	12,37	9,87	20,55	30,84	14,77	58,26	19,88	8,01	4,83	14,38	22,43	23,47	34,47	27,27	25,57
Nord estuary at 1000mt (sea water)	4,47	20,37	14,13	24,76	27,38	12,43	52,37	17,37	12,69	10,37	23,27	24,43	29,32	32,27	30,64	34,07
Nord estuary at 1500mt (sea water)	16,30	11,58	17,48	20,85	28,70	12,92	29,09	14,85	19,78	4,80	10,45	34,05	33,08	40,03	39,17	34,82
Central estuary at 500mt (sea water)	10,47	13,76	12,43	17,75	14,25	8,55	10,59	9,36	15,46	8,76	16,24	11,15	24,21	20,16	16,26	24,56
Central estuary at 1000mt (sea water)	10,51	25,47	27,22	31,86	29,37	21,58	32,70	22,00	21,66	4,34	3,75	11,34	41,85	5,73	13,86	25,47
Central estuary at 1500mt (sea water)	26,27	12,58	34,89	32,91	32,79	23,96	25,54	24,47	26,85	32,59	42,04	43,02	48,11	28,02	21,05	27,05
South estuary at 500mt (sea water)	23,79	22,34	31,76	32,37	34,38	22,76	28,22	27,48	23,35	23,55	34,48	36,57	47,47	37,39	40,24	38,07
South estuary at 1000mt (sea water)	16,54	10,26	33,12	31,71	35,47	26,48	32,85	28,04	29,54	21,76	20,57	19,76	44,22	36,17	40,27	40,28
South estuary at 1500mt (sea water)	18,47	11,35	30,48	30,57	34,12	24,83	34,47	26,57	30,57	36,47	37,27	44,27	46,37	46,35	45,97	47,67
**July**																
River Mouth (sea water)	6,11	18,87	13,45	11,75	20,43	17,48	22,47	18,26	12,12	15,57	12,26	21,87	11,87	22,69	23,69	27,26
Nord estuary at 500mt (sea water)	7,88	7,26	8,57	9,97	8,75	6,85	7,58	10,73	8,42	8,80	11,79	6,72	10,69	6,63	8,58	15,66
Nord estuary at 1000mt (sea water)	4,25	6,26	7,25	8,51	8,53	8,61	7,26	9,13	7,25	2,88	6,14	6,05	9,03	10,40	11,65	12,21
Nord estuary at 1500mt (sea water)	4,09	5,53	6,25	19,67	22,21	8,53	5,03	7,25	5,53	2,71	2,51	3,82	4,02	8,45	8,26	8,12
Central estuary at 500mt (sea water)	8,15	10,24	18,21	9,02	11,02	15,40	18,02	10,25	10,22	7,05	8,02	9,06	12,02	8,03	12,02	15,41
Central estuary at 1000mt (sea water)	10,42	9,05	17,67	10,27	19,46	13,91	16,48	13,05	13,82	9,05	10,05	11,27	14,05	19,05	14,36	12,75
Central estuary at 1500mt (sea water)	13,21	12,77	9,15	22,33	11,27	12,03	15,10	11,34	19,65	6,68	5,05	17,33	23,01	18,10	21,23	22,17
South estuary at 500mt (sea water)	7,24	24,95	22,74	23,91	22,43	17,33	23,60	21,26	15,03	9,56	10,26	13,44	16,06	6,75	13,03	14,33
South estuary at 1000mt (sea water)	5,25	14,97	19,74	17,06	18,82	15,85	13,05	9,86	15,13	10,02	19,23	28,05	39,05	22,04	24,02	22,12
South estuary at 1500mt (sea water)	16,30	23,58	17,48	20,85	28,70	12,92	29,09	14,85	19,78	4,80	15,45	34,05	33,08	40,03	39,17	37,22

Table S8. Description of concentration of PAHs in sediment samples of the Sele River, southern Italy.

	Nap	Acy	Ace	Flu	Phe	An	Fl	Pyr	BaA	Chr	BbF	BkF	BaP	DahA	BghiP	InP
River Mouth (sea water)	28,37	22,05	33,73	40,17	53,14	34,12	77,68	58,39	45,23	39,62	81,43	42,25	104,74	73,55	58,45	78,25
Nord estuary at 500mt (sea water)	8,11	26,62	19,92	27,22	30,30	30,71	39,30	20,35	24,37	29,60	87,49	87,31	89,66	107,35	68,63	15,52
Nord estuary at 1000mt (sea water)	8,72	31,99	22,72	26,90	31,17	29,99	34,88	23,79	20,56	32,88	69,73	70,32	84,00	66,22	49,34	46,39
Nord estuary at 1500mt (sea water)	7,81	10,88	12,18	15,70	18,52	16,93	16,42	8,19	19,38	14,68	36,88	36,00	47,04	52,99	11,29	6,86
Central estuary at 500mt (sea water)	73,78	14,47	17,67	16,38	15,22	9,81	5,23	12,11	30,81	27,90	81,76	87,68	149,56	98,77	49,21	33,95
Central estuary at 1000mt (sea water)	7,49	13,52	15,11	16,35	17,25	20,37	41,04	11,81	21,61	26,00	95,85	109,78	157,86	129,76	6,87	17,46
Central estuary at 1500mt (sea water)	6,79	11,85	8,20	9,90	36,06	9,87	48,94	9,91	16,82	14,53	94,20	90,63	129,61	98,44	10,89	5,54
South estuary at 500mt (sea water)	65,27	13,63	16,17	16,81	16,80	15,26	58,34	43,34	43,68	45,90	95,15	101,98	89,56	90,96	75,56	63,80
South estuary at 1000mt (sea water)	7,36	10,26	16,22	17,19	17,18	15,61	41,62	15,06	22,79	25,68	98,91	90,79	99,51	146,57	68,88	50,69
South estuary at 1500mt (sea water)	5,30	15,76	17,30	8,65	19,24	13,61	73,10	72,67	51,92	36,34	79,04	79,98	101,65	88,95	13,63	6,12

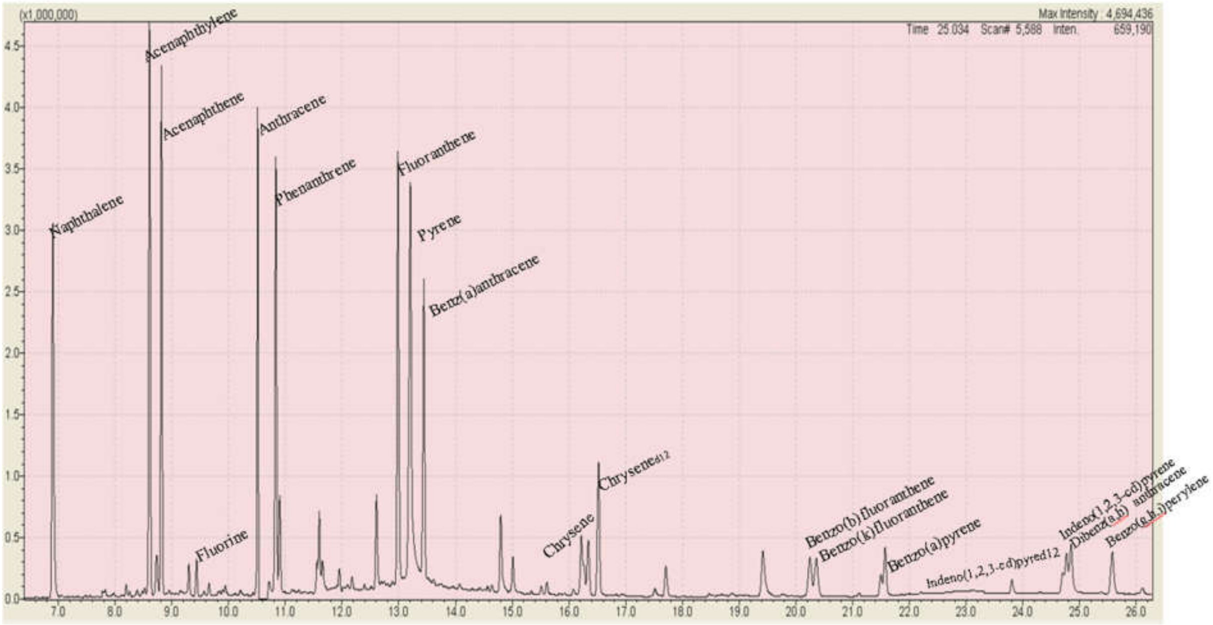

(**a**)

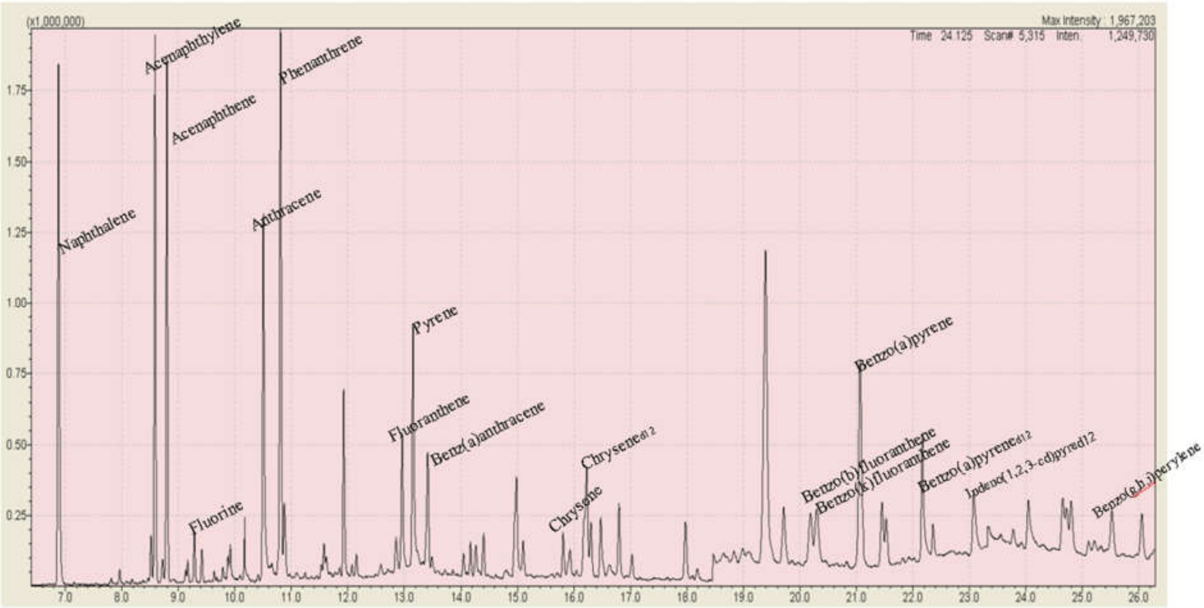
(x1,000,000)
Max Intensity : 1,967,203
Time 24.125 Scan# 5,315 Inten. 1,249,730
Naphthalene
Acenaphthylene
Acenaphthene
Anthracene
Phenanthrene
Fluorine
Pyrene
Fluoranthene
Benz(a)anthracene
Chrysene
Chrysened12
Benzo(a)pyrene
Benzo(b)fluoranthene
Benzo(k)fluoranthene
Benzo(a)pyrened12
Indeno(1,2,3-cd)pyred12
Benzo(g,h,i)perylene

(**b**)

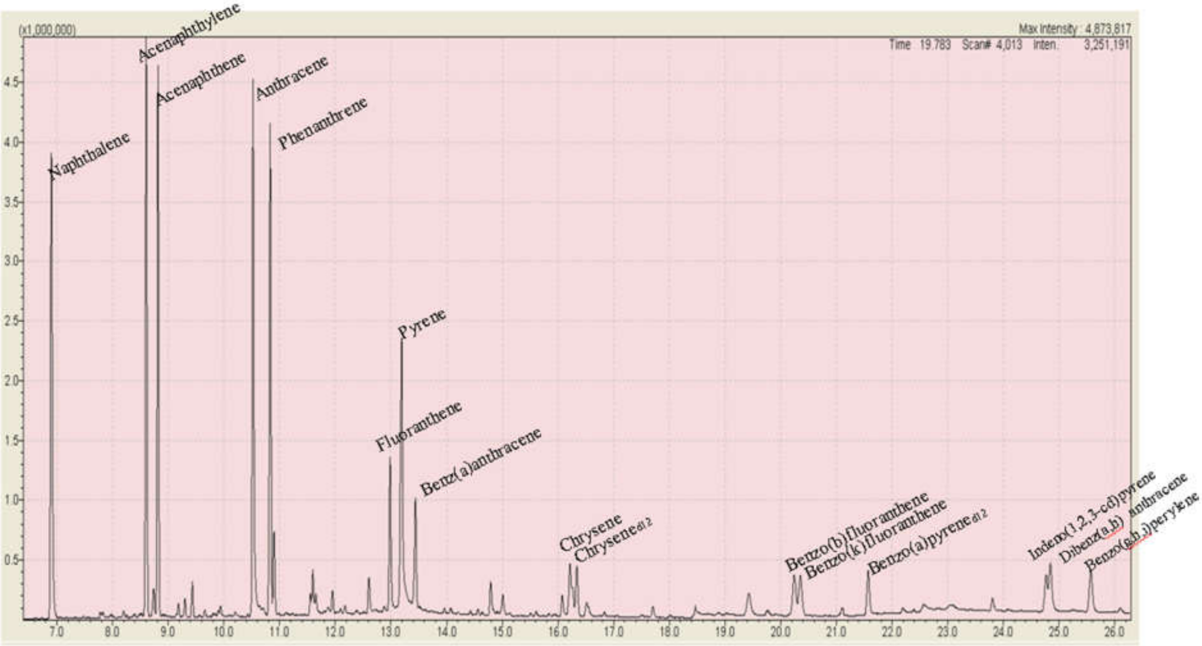
(x1,000,000)
Max Intensity : 4,873,817
Time 19.783 Scan# 4,013 Inten. 3,251,191
Naphthalene
Acenaphthylene
Acenaphthene
Anthracene
Phenanthrene
Pyrene
Fluoranthene
Benz(a)anthracene
Chrysene
Chrysened12
Benzo(b)fluoranthene
Benzo(k)fluoranthene
Benzo(a)pyrened12
Indeno(1,2,3-cd)pyrene
Dibenz(a,h) anthracene
Benzo(g,h,i)perylene

(**c**)

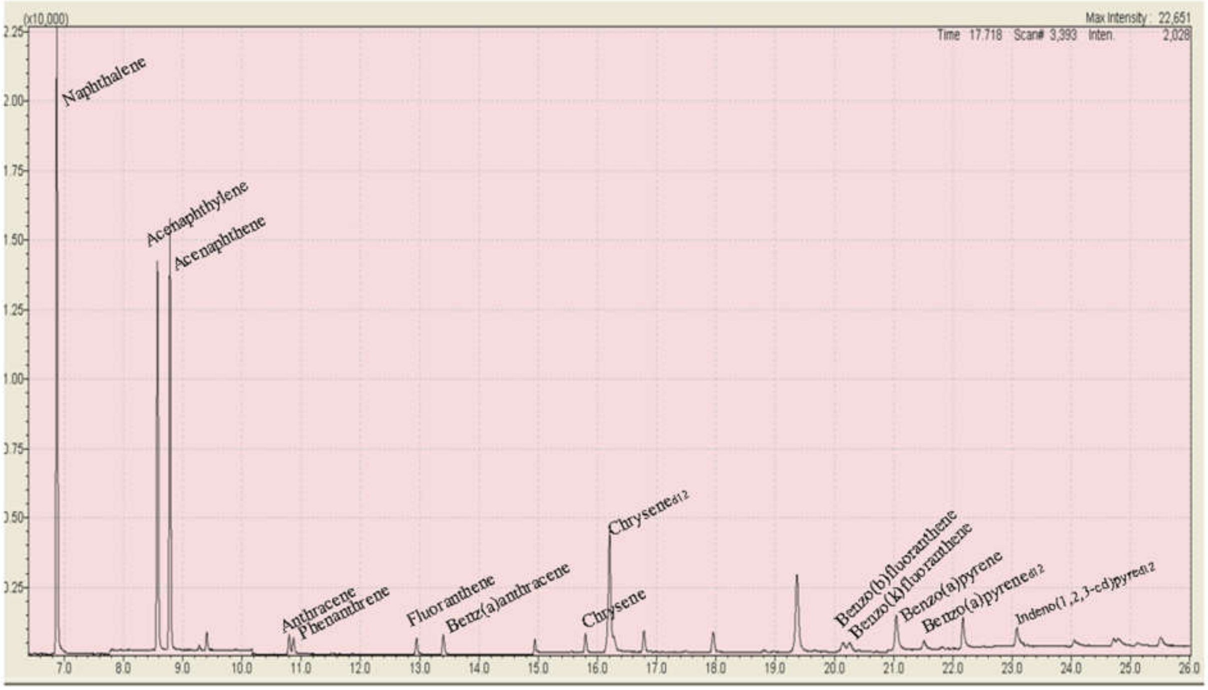

(x10,000)
Max Intensity : 22,651
Time 17.718 Scan# 3,393 Inten. 2,028
Naphthalene
Acenaphthylene
Acenaphthene
Anthracene
Phenanthrene
Fluoranthene
Benz(a)anthracene
Chrysened12
Chrysene
Benzo(b)fluoranthene
Benzo(k)fluoranthene
Benzo(a)pyrene
Benzo(a)pyrened12
Indeno(1,2,3-cd)pyred12

(**d**)

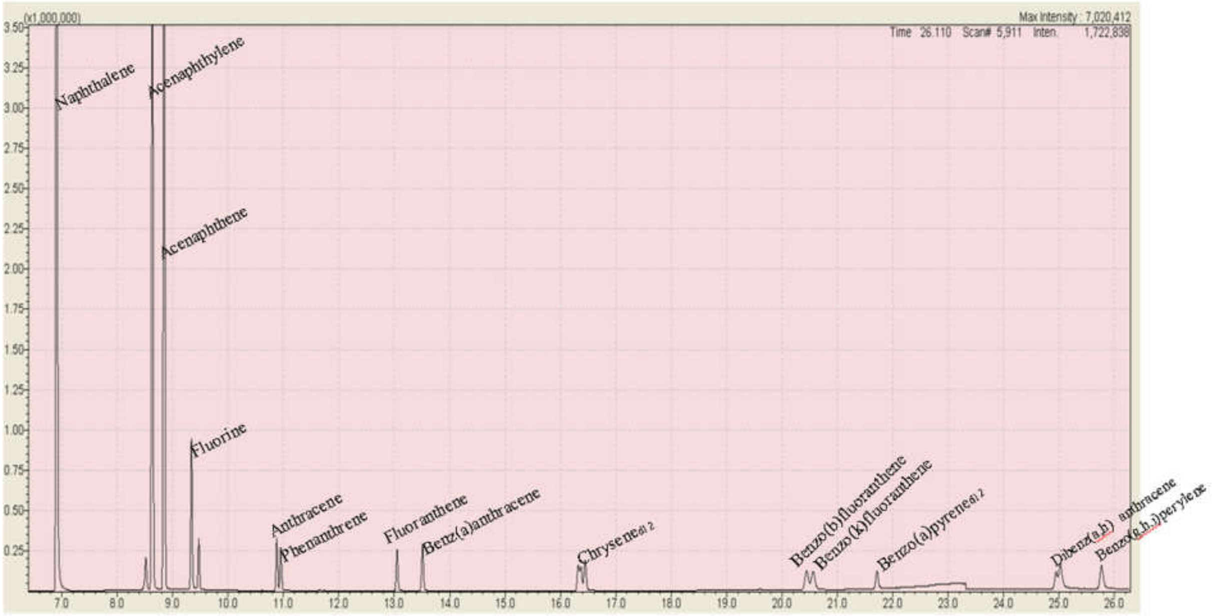

(x1,000,000)
Max Intensity : 7,020,412
Time 26.110 Scan# 5,911 Inten. 1,722,838
Naphthalene
Acenaphthylene
Acenaphthene
Fluorine
Anthracene
Phenanthrene
Fluoranthene
Benz(a)anthracene
Chrysened12
Benzo(b)fluoranthene
Benzo(k)fluoranthene
Benzo(a)pyrened12
Dibenz(a,h) anthracene
Benzo(g,h,i)perylene

(**e**)

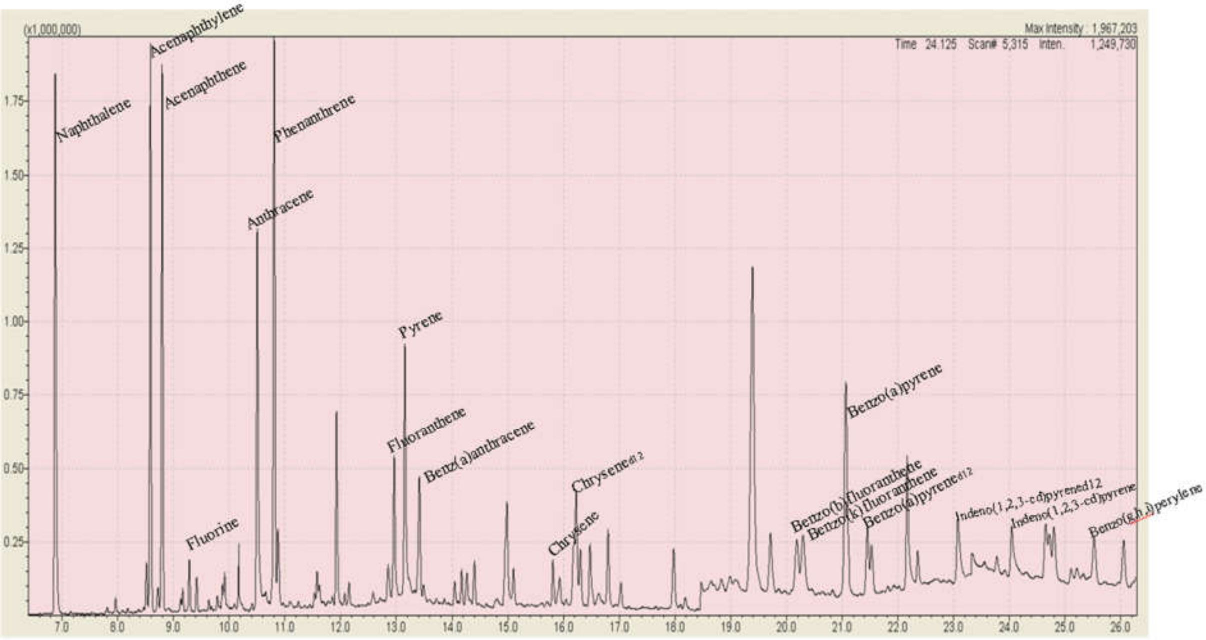

(**f**)

Figures S1. Chromatograms obtained in different phase of Sele River. (**a**) PAHs chromatogram identified in a water sample (Dissolved phase) of Sele River. (**b**) PAHs chromatogram identified in a Suspended particulate matter (SPM) sample of Sele River. (**c**) PAHs chromatogram identified in a water sample (Dissolved phase) of Sele River. (**d**) PAHs chromatogram identified in a Suspended particulate matter (SPM) sample of Sele River. (**e**) PAHs chromatogram identified in Sediment sample of Sele River. (**f**) PAHs chromatogram identified in Sediment sample of Sele River.
